# Relationships between the Nutrition Status and Oral Measurements for Sarcopenia in Older Japanese Adults

**DOI:** 10.3390/jcm11247382

**Published:** 2022-12-12

**Authors:** Kentaro Okuno, Ryuichiro Kobuchi, Suguru Morita, Ayako Masago, Masaaki Imaoka, Kazuya Takahashi

**Affiliations:** 1 Department of Geriatric Dentistry, Osaka Dental University, 1-8, Kuzuhahanazono-cho, Hirakata-shi 573-1121, Japan; 2 Department of Dentistry and Oral-Maxillofacial Surgery, Chikaishi Hospital, Medical Corporation Touhoukai, 2-46, Hikari-machi, Gifu-shi 502-0901, Japan

**Keywords:** nutrition, sarcopenia, tongue pressure

## Abstract

Introduction: The purpose of the present study was to clarify the relationships between the risk of malnutrition as a preliminary stage of malnutrition and overall and oral measurements for sarcopenia in older Japanese adults. Methods: Forty-five participants (79.7 ± 6.1 years) were included in the analysis. The nutrition status of the participants was assessed using the Mini Nutritional Assessment-Short Form (MNA-SF) and classified into two groups: normal and at risk of malnutrition. Overall measurements for sarcopenia in the present study were the skeletal muscle mass index, grip strength, and walking speed, while oral measurements were the cross-sectional area of the geniohyoid muscle, tongue pressure (TP), and oral diadochokinesis. Results: MNA-SF correlated with TP (r = 0.347, *p* = 0.019). We observed decreases of 5.7 kPa in TP and 3.9 kg/cm^2^ in BMI in the at risk of malnutrition group. A multiple regression analysis of parameters contributing to the risk of malnutrition identified TP as an independent variable (β = 0.913, *p* = 0.042). Conclusions: The present results demonstrate that the risk of malnutrition is associated with TP as an oral measurement for sarcopenia, but not overall measurements for sarcopenia. Therefore, low TP may be related with the risk of malnutrition.

## 1. Introduction

Older adults are at risk of malnutrition due to a decline in physiological function with aging and socioeconomic and psycho-behavioral factors [1]. Malnutrition is a risk factor for sarcopenia, which is characterized by the loss of skeletal muscle mass and strength, thereby reducing the basal metabolic rate in a negative chain reaction that lowers energy consumption and nutrient intake [2]. Sarcopenia may occur secondary to a systemic disease, particularly one that induces inflammatory processes (e.g., malignancy, inflammatory bowel diseases, malabsorption and malnutrition, physical inactivity, or organ failure). The emerging concept of the “gut –muscle axis” needs to be adequately described in the pathogenesis of sarcopenic dysphagia, taking into account the key role of inflammation and the gut microbiota in the development of muscle wasting [3]. The mechanisms underlying a low nutritional status involve a decline in oral function, which plays an important role in food intake; therefore, the deterioration of oral function in older adults worsens their nutritional status [4,5,6].

Among the tissues involved in oral function, the tongue plays an important role in swallowing and speech. Our research group examined the muscle mass, muscle strength, and motor function of the tongue in assessments of oral sarcopenia. Oral measurements for sarcopenia were identified as the cross-sectional area of the geniohyoid muscle (CSG), tongue pressure (TP), and oral diadochokinesis (ODK), and all endpoints of oral sarcopenia were influenced by overall measurements for sarcopenia [7].

Based on these findings, we hypothesized that oral measurements for sarcopenia may precede overall measurements for sarcopenia and contribute to the risk of malnutrition, the preliminary stage of malnutrition. The purpose of the present study was to clarify the relationships between the risk of malnutrition as a preliminary stage of malnutrition and overall and oral measurements for sarcopenia in older Japanese adults.

## 2. Materials and Methods

### 2.1. Participants

**Figure 1:**
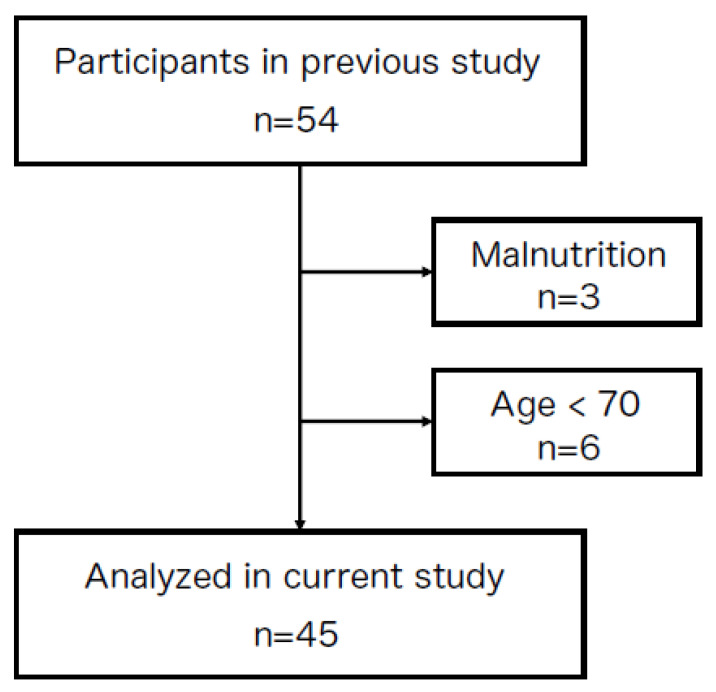
A diagram of study participants. Participants were selected from the database of our previous study [7]. Among 54 participants, 9 were excluded due to malnutrition and being younger than 70 years old; therefore, 45 participants were ultimately analyzed in the present study.

The present study was designed as a cross-sectional survey. Participants were selected from the database of our previous study [7]. Inclusion criteria were the nutrition status assessed by the Mini Nutritional Assessment-Short Form (MNA-SF) showing a normal status and risk of malnutrition. Exclusion criteria were age < 70 years, pacemaker wearers, non-ambulatory patients, an inability to communicate, and a malnutrition status assessed by MNA-SF. We calculated sample size based on the correlation between MNA-SF and tongue power in a previous study [7]. Twenty-six participants were required for 80% power, with an effect size of 0.5, with a two-sided alfa level of 0.05, for correlation (G*Power 3.1, Heinrich-Heine- Universität, Düsseldorf, Germany). Fifty-four participants from the database of our previous study were candidates for the present study. Three participants were excluded due to malnutrition and six for being younger than 70 years old. Therefore, 45 participants were ultimately analyzed in the present study (Figure 1). The Ethics Committee of Osaka Dental University approved the present study (Approval No. 110970).

### 2.2. Basic Information

Interviews with participants and clinical records provided information on age, sex, basic diseases, medical history, and medications. Body mass index (BMI) was calculated as weight divided by height squared. The Barthel Index was used to assess basic daily activities and, thus, functional independence in daily life [8]. Interviews with participants using the questionnaire on Eating Assessment Tool-10 (EAT-10) [9] were used to evaluate eating and swallowing functions.

### 2.3. Nutritional Risk Assessment

Interviews with participants using MNA-SF [10] were conducted to evaluate their nutritional status. MNA-SF includes the following six domains: appetite loss (0–2 points), weight loss (0–3 points), mobility (0–2 points), stress/acute disease (0 or 2 points), neuropsychological impairment (0–2 points), and BMI (0–3 points). If it was not possible to weigh a patient, calf circumference was used as a substitute for BMI (0 or 3 points). According to the total score of MNA-SF, patients were classified into three categories: malnourished (0–7 points), at risk of malnutrition (8–11 points), or a normal nutritional status (12–14 points).

### 2.4. Overall Measurements for Sarcopenia

A bioelectrical impedance analysis of skeletal muscle masses in the extremities was performed and used to calculate the skeletal muscle mass index (SMI). In Body S10 (In Body Japan, Tokyo, Japan) was used for these measurements, which were conducted in a standing position with electrodes attached to the thumbs and middle fingers of both hands and ankles of both legs. Patients were asked to remain quiet and still during measurements. Standard values for SMI reductions in males and females were previously reported to be ≤7.0 and <5.4 kg/m^2^, respectively [11].

A digital grip strength tester (Takei Scientific Instruments Co., Ltd., Yashiroda Akiha-Ku Niigata City, Japan) was employed to assess grip strength (GS). The second joint of the index finger was adjusted at a right angle to the grip of the strength tester, and participants held the tester without shaking their arm. Measurements were conducted in duplicate alternating between the left and right hands, and the maximum value was used as GS. Standard values for GS reductions in males and females were previously reported to be <26 and <18 kgf, respectively [11].

Walking speed (WS) was assessed over a distance of 9 m, which comprised 2 m at the start and end points as a preparatory route and 5 m for the measurement of WS [12]. Participants were asked to walk as usual without knowledge of the measurement being performed. The start and 2-, 7-, and 9 m (goal) points were marked. The measurement started when one foot touched the 2 m point and ended when one foot touched the 7 m point. The calculation of WS involved dividing 5 m by the measured time. Measurements were conducted in duplicate, with the faster WS being used as the measured value. The standard value for a WS reduction in both males and females was previously reported to be <0.8 m/s [11].

### 2.5. Oral Measurements for Sarcopenia

CSG, a muscle that plays a role in swallowing function [13], was evaluated as an indicator of muscle mass in oral measurements for sarcopenia. An ultrasonic diagnostic system was employed to examine the geniohyoid muscle [14]. Each participant was seated in a comfortable posture on a reclining chair at an angle of 30° from the floor. A 3.5 MHz convex probe ultrasonic diagnostic system (Miruco^®^; Nippon Sigmax Co., Ltd., Tokyo, Japan) was attached perpendicularly to the lower surface of the geniohyoid muscle in the center of the mouth floor such that it did not touch thyroid cartilage and its surface was in close contact with the bottom of the jaw. Ultrasonic gel was used at an amount that did not compress soft tissue under the probe. The hyoid bone and mandible with acoustic shadows and the geniohyoid muscle attached to both were shown in the B-mode sagittal cross-sectional plane (frequency: 3.5 MHz) on a single screen, and the resting state was saved as a still image. Ultrasound images stored in the ultrasound diagnostic device were loaded onto a personal computer. The geniohyoid muscle depicted between the mandible and hyoid bone on ultrasound images was plotted using Image J software, followed by evaluations of the surrounding area. Ultrasonography and CSG measurements were conducted by the same examiner (RK).

A JMS tongue-measuring instrument was used to assess TP as an indicator of muscle strength in oral measurements for sarcopenia [15]. Following a calibration, the upper and lower front teeth held the concave part of the TP probe in place, while the examiner held the probe. Measurements were conducted as previously reported. After sufficient training, each participant pressed and crushed the vinyl bulge at the tip of the probe against the palate with maximum tongue force. Measurements were conducted in triplicate with a sufficient rest period between measurements. The average of three measurements was used as TP.

ODK is an indicator of motor function in oral measurements for sarcopenia [16]. The task performed by participants involved pronouncing /ta/ accompanied by the movement of the tongue tip. Each participant repeated the task as rapidly as possible in five seconds and took breaths where needed. Measurements were conducted in triplicate using the calculator method with a sufficient rest period between measurements. The ODK value of /ta/ was calculated as the mean number of pronunciations per second in three measurements.

### 2.6. Statistical Analysis

MNA-SF, SMI, GS, WS, CSG, TP, and ODK were assessed by Pearson’s correlation coefficient because of normal distributions using the Shapiro–Wilk test. According to the MNA-SF classification, participants were classified into the at risk of malnutrition group and normal nutritional status group. The *t*-test was used to investigate the significance of differences in age, BMI, SMI, GS, WS, CSG, TP, and ODK between the normal status group and the at risk of malnutrition group (significance level: <0.05). A multivariable logistic regression analysis was conducted with the risk of malnutrition as the dependent variable and SMI, GS, WS, CSG, TP, and ODK as independent variables (significance level: <0.05). A stepwise forward selection procedure was also used to assess the effects of different variables and identify important explanatory variables. Statistical analyses were conducted using SPSS Statistics 22 (IBM, Armonk, NY, USA).

## 3. Results

The data of 45 participants (14 males and 31 females, mean age of 79.7 ± 6.1 years) were analyzed in the present study. Table 1 shows basic information on each participant. Mean BMI was 21.9 ± 3.2 kg/cm^2^, which was within the normal range. The mean Barthel Index (99.1 ± 2.9) was high, indicating that participants were living independently. Mean values for overall and oral measurements for sarcopenia were within standard values. Muscle mass and strength as well as motor function were assessed in relation to overall and oral measurements for sarcopenia.

Table 2 shows the relationships among MNA-SF, SMI, GS, WS, CSG, TP, and ODK. TP strength was 0.347 higher with each unit of increase in the value for MNA-SF (r = 0.347, *p* = 0.019). In terms of the relationship between overall and oral measurements for sarcopenia, a correlation was noted between SMI and CSG (r = 0.529, *p* = 0.000), GS and TP (r = 0.422, *p* = 0.004), and WS and ODK (r = 0.531, *p* = 0.000). A moderate correlation was found between overall and oral measurements for sarcopenia.

Table 3 shows comparisons of age, BMI, and the parameters of overall and oral measurements for sarcopenia between the normal nutrition and at risk of malnutrition groups. We observed decreases of 5.7 kPa in TP and 3.9 kg/cm^2^ in BMI in the at risk of malnutrition group (BMI: 23.8 ± 2.1 kg/cm^2^ vs. 19.9 ± 3.0 kg/cm^2^, *p* = 0.000; TP: 30.6 ± 7.6 kPa vs. 25.9 ± 7.0 kPa, *p* = 0.035). Participants in the at risk of malnutrition group were slightly thin and had low tongue strength. There were no correlations among the parameters of overall measurements for sarcopenia (SMI, GS, and WS) between the groups.

Parameters contributing to the risk of malnutrition were examined using a multiple regression analysis, which was conducted with the risk of malnutrition as the dependent variable and overall (SMI, GS, and WS) and oral (CSG, TP, and ODK) measurements for sarcopenia as independent variables. The results obtained identified TP as an independent variable (β = 0.913, *p* = 0.042) (Table 4). Among the parameters of overall and oral measurements for sarcopenia examined, only TP was associated with the risk of malnutrition.

## 4. Discussion

Basic information and the endpoints of overall and oral measurements for sarcopenia were investigated in the present study to elucidate their relationships with the nutrition status in older community-dwelling individuals. The results obtained revealed a correlation between MNA-SF as a diagnostic index of the nutrition status and TP as an oral measurement for sarcopenia. TP was 5.7 kPa lower in the at risk of malnutrition group than in the normal nutrition group. There were no correlations among the parameters of overall measurements for sarcopenia between the normal nutrition and at risk of malnutrition groups.

Malnutrition is a risk factor for sarcopenia because a decrease in muscle mass reduces the basal metabolic rate in a negative chain reaction that results in lower energy consumption and lower nutrient intake [2]. Demographic and socioeconomic factors, psycho-behavioral factors, and the nutritional status accelerate this negative chain, which a previous study termed the cycle of frailty [17]. Sarcopenia is considered ‘primary’ (or age-related) when no other specific cause is evident, and ‘secondary’ when causal factors other than aging are present. Sarcopenia may occur secondary to a systemic disease, particularly one that induces inflammatory processes (e.g., malignancy, inflammatory bowel diseases, malabsorption and malnutrition, physical inactivity, or organ failure). The emerging concept of the “gut–muscle axis” needs to be adequately described in the pathogenesis of sarcopenic dysphagia, taking into account the key role of inflammation and the gut microbiota in the development of muscle wasting [3]. In the present study, we focused on the status of the risk of malnutrition.

Parameters between overall and oral measurements for sarcopenia moderately correlated. SMI, an indicator of muscle mass in overall measurements for sarcopenia, correlated with CSG, an indicator of muscle mass in oral measurements for sarcopenia, TP, an indicator of muscle strength in oral measurements for sarcopenia, correlated with ODK, an indicator of muscle dexterity measurements implying neurological integrity in oral measurements for sarcopenia. This result showed the relevance between overall and oral measurements for sarcopenia, which is consistent with our previous findings [7].

A relationship has been reported between oral function and the nutrition status. An analysis of more than 5000 Japanese older adults revealed that TP decreased with advancing age in both males and females [18]. Mean (years) TP measurements in male participants were 34.0 (65–69), 32.2 (70–74), 30.8 (75–79), 28.4 (80–84), and 24.4 (≥85) kPa, while the corresponding values in female participants were 31.5 (65–69), 30.5 (70–74), 29.6 (75–79), 28.4 (80–84), and 26.4 (≥85) kPa. In the present study, the mean age was 79 years and mean TP was 28.3 kPa in both sexes, which were consistent with previously reported values [17].

In terms of TP in patients with dysphagia, mean TP with a dysphagia diet in patients with acute stroke was 21.7 kPa [19]. However, Maeda et al. [20] reported that the cut-off value of TP for decreased swallowing function was lower than 20 kPa because mean TP was 25.3 kPa in patients without dysphagia and 14.7 kPa in those with dysphagia. These findings suggest that the nutrition status is worse in patients with dysphagia; therefore, these patients may have a malnutrition status. In the present study, participants in the at risk of malnutrition group had an EAT-10 score of 1.9, which was below the cut-off value and, thus, they were a population without dysphagia. TP in the risk of malnutrition group in the present study—which was 25.9 kPa and, thus, was slightly higher than that in groups with dysphagia in previous studies—was appropriate for a population at risk of malnutrition.

A relationship was previously reported between the risk of malnutrition and TP in community-dwelling older individuals in Taiwan [21]. TP positively correlated with MNA scores and did not significantly differ between the normal nutrition and at risk of malnutrition groups. However, when participants were divided into four subgroups based on the quartiles of TP and age and sex were adjusted for, the subgroup in the third quartile had a significantly higher risk (OR = 4.85) of malnutrition. The present study also demonstrated that TP correlated with MNA-SF (r = 0.347), which was smaller in the at risk of malnutrition group (TP: 30.6 ± 7.6 kPa vs. 25.9 ± 7.0 kPa), and this was consistent with previous findings. Expect for TP, the correlations between other parameters (CSG, ODK, SMI, GF, and WS) and the MNA-SF score were weak. The nutrition status of participants in the present study was either normal or at risk of malnutrition, and participants with malnutrition were excluded because the focus of the present study was the risk of malnutrition as a preliminary stage of malnutrition. This may be the reason for the weak correlation with the MNA-SF score and is a limitation of the present study.

Previous studies reported a relationship between oral function and CSG. Shimizu et al. [22] measured the mass of the geniohyoid muscle in perioperative patients using ultrasonography. The findings obtained revealed significant reductions in the area of the geniohyoid muscle after surgery (preoperative = 203 mm^2^, postoperative day 7 = 176 mm^2^, and postoperative day 14 = 174 mm^2^). Moreover, the postoperative percent decrease in the area of the geniohyoid muscle was greater on average in the poor oral intake group than in the good oral intake group, and a significant difference was observed on postoperative day 14. Patient characteristics, namely, a mean age of 70.6 years and BMI of 21.2 kg/cm^2^, were similar to those of the participants in the present study. However, the MNA-sf score in the previous study was 7 points, which is borderline between malnutrition, and lower than that (11.6 points) in the present study. Patients had esophageal, gastric, small bowel, or colon cancer. The populations in the previous and present studies appeared to be similar before surgery and, thus, the area of the geniohyoid muscle was also similar (203 mm^2^ preoperatively in the Shimizu study [21] vs. 223.3 mm^2^ in the at risk of malnutrition group in the present study). Another study [23] reported the areas of the geniohyoid muscle as 136.3 mm^2^ in patients with sarcopenic dysphagia and 154.4 mm^2^ in those without. The cut-off value to detect sarcopenic dysphagia was previously reported to be 116.6 mm^2^. The population of the previous study was older and slightly thinner, with a mean age of 82.1 years and BMI of 20.0 kg/cm^2^, than the participants in the present study, and included patients with dysphagia. Therefore, participants in the present study were healthier older adults than those in previous studies. The area of the geniohyoid muscle was 223.3 mm^2^ in the at risk of malnutrition group in the present study, which was an appropriate value for a population at risk of malnutrition.

In the present study, TP, the indicator of muscle strength in oral measurements for sarcopenia, was significantly smaller in the at risk of malnutrition group, whereas the parameters of overall measurements for sarcopenia (SMI, GS, and WS) did not significantly differ between the groups. Participants in the at risk of malnutrition group were slightly thinner and had low tongue strength. There were no correlations among the parameters of overall measurements for sarcopenia between the groups. A multiple regression analysis of the parameters contributing to the risk of malnutrition was conducted and identified TP as a significant factor (β = 0.913, *p* = 0.042), but not other parameters, including SMI, GS, WS, CSG, and ODK. These results suggest a relationship between the risk of malnutrition and TP in older adults as oral measurements for sarcopenia, but not overall measurements for sarcopenia. Weak tongue strength may be associated with the hypoactivity of masticatory muscles and affect the food intake balance, thereby increasing the risk of malnutrition, the preliminary stage of malnutrition.

There are a number of limitations in this study that need to be addressed. Since this was a cross-sectional analysis, a prospective cohort study on the causal relationship between TP and malnutrition that ideally includes a malnutrition group is needed. In the present study, the majority of participants lived independently; therefore, they were in the state of normal nutrition or at risk of malnutrition, while few had malnutrition. Furthermore, we did not perform a sex-specific analysis because the sample size was small. The feature of the nutrition status and the standard values for muscle mass and strength differed between males and females. As previously mentioned, the sample size of the present study was small. Regarding the correlation, twenty-six participants were required based on calculation software and a requisite number of participants was obtained for this study. However, in regard to the *t*-test between two groups, the sample size of the present study was small, because 128 patients were required according to the software. This suggests the possibility that in larger studies other parameters may display significant differences, especially walking speed (*p* = 0.054). In the future, a large number of participants, including the same number of males and females, and multilevel states of nutrition (normal, at risk of malnutrition, and malnutrition) need to be examined in a prospective cohort study.

## 5. Conclusions

The present study demonstrated that the risk of malnutrition was associated with TP in older adults as oral measurements for sarcopenia, but not overall measurements for sarcopenia. Therefore, low TP may be related with the risk of malnutrition, the preliminary stage of malnutrition.

## Figures and Tables

**Table 1:** Characteristics of the study population.

Variable	All (*n* = 45)
Age (years)	79.7 ± 6.1
Sex (%)	Male (31.1) Female (68.9)
BMI (kg/m^2^)	21.9 ± 3.2
BI	99.1 ± 2.9
MNA-sf	11.6 ± 1.8
EAT-10	2.2 ± 3.1
SMI (kg/m^2^)	6.1 ± 1.1
GS (kgf)	22.6 ± 6.9
WS (m/s)	1.03 ± 0.28
CSG (mm^2^)	235.2 ± 60.5
TP (Kpa)	28.3 ± 7.6
ODK (/s)	5.9 ± 0.8

Data are shown as the mean ± SD. BMI: body mass index; BI: Barthel index; MNA-sf: Mini Nutritional Assessment-Short Form; EAT-10: eating assessment tool-10; SMI: skeletal muscle mass index; GS: grip strength; WS: walking speed; CSG: cross-sectional area of the geniohyoid muscle; TP: tongue pressure; ODK: oral diadochokinesis of /ta/.

**Table 2:** Bivariate simple correlation analysis of MNA-sf and overall and oral measurements for sarcopenia.

*n* = 45	ODK	TP	CSG	WS	GF	SMI	MNA-sf
MNA-sf	0.278	0.347 *	0.091	0.264	0.126	0.224	1
SMI	0.384 **	0.411 **	0.529 **	0.278	0.793 **	1	
GS	0.337 *	0.422 **	0.335 *	0.293	1		
WS	0.531 **	0.475 **	0.116	1			
CSG	0.344 *	0.498 **	1				
TP	0.420 **	1					
ODK	1						

Coefficients of correlation were calculated with Pearson’s product moment correlation coefficient for parametric values. ** *p* < 0.01 * *p* < 0.05. MNA-sf: Mini Nutritional Assessment-Short Form; SMI: skeletal muscle mass index; GS: grip strength; WS: walking speed; CSG: cross-sectional area of the geniohyoid muscle; TP: tongue pressure; ODK: oral diadochokinesis of /ta/.

**Table 3:** Comparisons of overall and oral measurements for sarcopenia between the normal and at risk of malnutrition groups.

	Normal	At Risk of Malnutrition	*p*
*n*	23	22	
Age	80.0 ± 6.4	79.5 ± 6.0	0.786
Sex %Male	26.0%	36.0%	0.468
BMI	23.8 ± 2.1	19.9 ± 3.0	0.000
SMI	6.3 ± 1.0	5.8 ± 1.0	0.112
GS	23.4 ± 6.8	21.7 ± 7.0	0.414
WS	1.1 ± 0.2	0.9 ± 0.3	0.054
CSG	246.5 ± 66.2	223.3 ± 52.9	0.202
TP	30.6 ± 7.6	25.9 ± 7.0	0.035
ODK	6.0 ± 0.9	586 ± 0.8	0.278

The *t*-test. BMI: body mass index; SMI: skeletal muscle mass index; GS: grip strength; WS: walking speed; CSG: cross-sectional area of the geniohyoid muscle; TP: tongue pressure; ODK: oral diadochokinesis of /ta/.

**Table 4:** Multiple regression analysis of the risk of malnutrition.

Independent Variable	β	Wald	*p*
Tongue pressure	0.913	4.117	0.042

## Data Availability

The data presented in this study are available on request from the corresponding author. The data are not publicly available due to ethical restrictions.
